# Effect of Temperature on Carbapenemase-Encoding Plasmid Transfer in *Klebsiella pneumoniae*

**DOI:** 10.3390/microorganisms12030454

**Published:** 2024-02-23

**Authors:** Ji Woo Yang, Ji-Hyun Nam, Kwang Jun Lee, Jung Sik Yoo

**Affiliations:** 1 Division of Antimicrobial Resistance Research, National Institute of Health, Korea Disease Control and Prevention Agency, 187 Osongsaengmyeong 2-ro, Osong-eup, Heungdeok-gu, Cheongju-si 28159, Republic of Korea; gosky0127@korea.kr (J.W.Y.); wlgus829@korea.kr (J.-H.N.); 2 Division of Zoonotic and Vector Borne Disease Research, National Institute of Health, Korea Disease Control and Prevention Agency, 220 Osongsaengmyeong 2-ro, Osong-eup, Heungdeok-gu, Cheongju-si 28160, Republic of Korea; kwangjun@korea.kr

**Keywords:** antibiotic resistance, carbapenemase-producing *Enterobacterales*, *Klebsiella pneumoniae*, plasmid transfer, temperature

## Abstract

Bacteria causing human infections can develop antibiotic resistance due to various factors. Temperature affects bacterial growth and gene transfer; however, studies exploring the association between the changes in local temperature and antibiotic resistance are limited. Here, we investigated the effects of local temperatures on the distribution of antibiotic resistance and transmission of carbapenemase-producing *Enterobacterales* using the data on Klebsiella pneumoniae from sentinel hospitals in eight regions included in the Korea Global Antimicrobial Resistance Surveillance System between 2017 and 2021. The resistance rates to most antibiotics, including carbapenems, varied significantly according to local temperature (*p* < 0.047), except for aminoglycosides. Conjugation experiments at various temperatures for strains encoding the carbapenemase gene on a plasmid revealed significant variation in the optimal conjugation temperatures for plasmids carrying *bla*_KPC_ and *bla*_NDM_ genes. The optimal conjugation temperatures demonstrating the highest stability for *bla*_KPC_- and *bla*_NDM_-carrying plasmids were 25 °C (*p* = 0.030) and 30 °C (*p* = 0.007), respectively. The stability of *bla*_KPC_-IncF was higher at 25 °C than that at 30 °C (*p* = 0.032) or 37 °C (*p* = 0.047), while *bla*_KPC_-IncX3 exhibited the lowest stability at 37 °C (*p* = 0.047). *bla*_NDM_-IncX3 was more stable at 30 °C than at 37 °C (*p* = 0.049). These findings suggest that the optimal temperature for carbapenemase gene transmission varied between 25 °C and 30 °C, indicating that warmer seasons promote the transfer of more antibiotic resistance-related genes and highlighting the importance of local temperature in the spread and transmission of plasmids carrying carbapenemases.

## 1. Introduction

Antibiotic resistance poses a significant global public health threat and is anticipated to accelerate the development of resistance to current antibiotics [1]. Several studies have investigated antibiotic resistance, identifying various underlying factors; however, the effects of climate change, which refers to increasing changes in temperatures, remain poorly understood [2,3,4,5,6]. The World Health Organization (WHO) confirmed the important impact of warming on various infectious diseases worldwide. Temperature is a key factor affecting bacterial survival in the presence of antibiotics, and environmental traits are drastically increasing due to climate change [6]. Temperature affects bacterial growth in vitro and modulates the transfer of genomic material, including genes that encode (or confer) antibiotic resistance [3]. Several environmental factors, such as air, water, temperature, and food, are associated with infections and disease occurrence, with seasonal changes being particularly important [4]. Seasonal patterns have been observed for Gram-negative infections in bloodborne diseases, with peak infection rates occurring in the summer and correlating with increasing temperatures. In contrast, the resistance rate to Gram-positive infections has been reported to decrease with increasing temperatures [2,4,5]. These findings underscore the importance of temperature in the dissemination of antibiotic resistance; however, precise knowledge of their exact effects is lacking.

The WHO has declared CRE to be one of the most critical antibiotic-resistant pathogens. Carbapenem-resistant *Enterobacterales* (CRE) are usually multidrug-resistant and are often associated with treatment failure [1,6]. In particular, the increase in carbapenem resistance caused by the global spread of carbapenemase-producing *Enterobacterales* (CPE) is of great concern [7]. Carbapenem resistance in clinical strains is mediated by carbapenemases encoded by various plasmids [7]. Carbapenemases are beta-lactamases, including *Klebsiella pneumoniae* carbapenemase (KPC), New Delhi metallo-β-lactamase (NDM), oxacillinase-48 (OXA-48), Verona integron-encoded metallo-β-lactamase (VIM), and imipenemase (IMP), which hydrolyze penicillins, cephalosporins, monobactams, and carbapenems, rendering them ineffective [8]. Among these Carbapenemases, KPC causes serious nosocomial infections in patients with compromised immune systems, resulting in high mortality rates. According to the 2022 WHO GLASS report, more than 8% of bloodstream infections caused by *K. pneumoniae* showed carbapenem resistance. Despite extensive research to understand the resistance and dissemination mechanisms of antibiotic-resistant bacteria, such as CRE, their occurrence is increasing annually [9]. KPC possesses an excellent ability to acquire mobile genetic elements that encode multidrug resistance and high pathogenicity [7]. Among the known groups of genes encoding carbapenemase enzymes, *bla*_KPC_ and *bla*_NDM_ are the most prevalent, and the co-occurrence of factors conferring multiple resistance has been frequently reported [9]. It has been reported that the frequent exchange of plasmids carrying carbapenemase genes among strains increases the risk of CRE infection [9]. Additionally, environmental factors, such as increased temperature, have been shown to affect the prevalence of CPE [8]. However, studies investigating the effects of increased warming on antibiotic resistance in CPE are limited.

In this study, we aimed to investigate the effects of local temperature on the distribution of antibiotic resistance in *K. pneumoniae* and the spread of plasmids carrying carbapenemase genes. The findings of this study will contribute to a better understanding of the factors that contribute to antibiotic resistance.

## 2. Materials and Methods 

### 2.1. Data Sources and Collection

Korea Global Antimicrobial Resistance Surveillance System (Kor-GLASS) is an antimicrobial resistance (AMR) surveillance system that was established by the Korea Disease Control and Prevention Agency (KDCA) in 2016. It provides data for representative non-duplicate clinical isolates of major pathogens collected from sentinel hospitals in eight regions across the Korean Peninsula, along with patient clinical data [10]. In this study, we obtained data on the origin of infection and antibiotic resistance in *K. pneumoniae* included in Kor-GLASS between January 2017 and December 2021. Infection origin was categorized according to the number of hospitalization days at the time of specimen sampling. The monthly average temperature of each region was collected from the Korea Meteorological Administration (http://data.kma.go.kr, accessed on 7 April 2023). We divided the temperature ranges of the eight regions into four groups, <0 °C, 0–10 °C, 10–20 °C, and >20 °C, to analyze the correlation between local temperature and antibiotic resistance rates.

### 2.2. Bacterial Isolates and Detection of CPE

CPE were collected from 33 general hospitals in the National Laboratory Surveillance System of the KDCA. A total of 2186 strains of *K. pneumoniae* were collected between 2011 and 2015, of which 749 were CPE. The isolates were identified using a Bruker MALDI-TOF MS instrument and 16S rDNA sequencing performed at a national reference laboratory [10]. Antibiotic susceptibility testing (AST) was performed according to minimum inhibitory concentration (MIC) via broth microdilution, following the protocol of the Clinical and Laboratory Standards Institute [11]. All carbapenem-resistant strains were tested for carbapenemase gene presence, as previously reported [12].

### 2.3. PCR-Based Replicon Typing (PBRT) and Multilocus Sequence Typing (MLST)

Plasmid characterization of donors and transconjugants was performed with PBRT using a PBRT kit 2.0 (Diatheva, Fano, Italy). This system, consisting of eight multiplex PCR assays, allows for the identification of the following 30 replicons found in the *Enterobacterales* family: HI1, HI2, I1, I2, X1, X2, X3, X4, L, M, N, FIA, FIB, FIC, FII, FIIS, FIIK, FIB KN, FIB KQ, W, Y, P1, A/C, T, K, U, R, B/O, HIB-M, and FIB-M. All PCRs were performed according to the manufacturer’s instructions and included positive controls [9].

Seven targeted housekeeping genes (*gapA*, *infB*, *mdh*, *pgi*, *phoE*, *rpoB*, and *tonB*) were amplified according to Protocol 2 of the MLST Institute Pasteur database (https://bigsdb.pasteur.fr/, accessed on 11 October 2022). This protocol uses primers with universal sequencing tails to amplify all genes at the same temperature and sequences them using the same forward and reverse primers [9].

### 2.4. Plasmid Transfer by Bacterial Conjugation

Transconjugants were obtained using carbapenemase-producing *K. pneumoniae* (CP-KP) strains as donors and sodium azide-resistant *Escherichia coli* J53 as recipients. Equal amounts of exponential cultures of the donor and recipient strains were mixed, incubated in Mueller–Hinton broth for 12 h, and spread on brain–heart infusion agar containing sodium azide (10 mg/L) and ceftazidime (1 mg/L). The conjugation frequency according to temperature was measured at 20 °C, 25 °C, 30 °C, 37 °C, or 41 °C. The presence of CPE was confirmed for each colony using PCR [12]. Plasmid transfer frequency was calculated based on the number of transconjugants per donor [13]. Conjugation efficiency was measured at least three times for each strain. Experimental data are expressed as the mean ± standard deviation [14].

### 2.5. Confirmation of Plasmid Stability

A single colony of each transconjugant was cultured overnight in Luria–Bertani (LB) broth without antibiotics. Overnight-cultured bacteria were diluted to 1:100 in 10 mL of fresh LB broth; dilution and serial passaging were conducted in a similar manner every 12 h for 120 h. Cultures collected at each step were diluted to 10^−4^–10^−6^ after a 10-fold serial dilution. The cells were spread on MH plates with or without meropenem (1 μg/mL). Colony-forming units (CFUs) from plates containing antibiotics were considered to be plasmid-positive colonies, whereas CFUs from antibiotic-free plates were considered to be the total population in the culture. Plasmid frequency was determined by dividing the plasmid-positive CFUs by the total population CFUs and was representative of the plasmid stability of passaged transconjugants of each type: higher plasmid frequency suggests higher plasmid stability in the host [14].

### 2.6. Statistical Analysis

All data were analyzed using SPSS (version 29.0; IBM SPSS, Armonk, NY, USA). Chi-square tests were used to analyze the correlation between temperature and antibiotic resistance. Conjugation frequency and plasmid stability were tested using a *t*-test and analysis of variance (ANOVA), with statistical significance set at *p* < 0.05. The figures were constructed using GraphPad Prism (version 8; GraphPad Software, San Diego, CA, USA) [13,14,15].

## 3. Results

### 3.1. Antibiotic Resistance Rates of K. pneumoniae According to Local Temperature

We analyzed the antibiotic resistance rate of *K. pneumoniae* using data from Kor-GLASS. Between January 2017 and December 2021, a total of 9981 cases of *K. pneumoniae* infections were reported in blood and urine samples from sentinel hospitals in eight regions. Over 5 years, the average monthly temperature in areas from where samples were collected ranged from −4 °C to 29 °C. Significant differences were observed for each temperature group in all antibiotic classes (*p* < 0.047) except aminoglycosides (*p* = 0.492; Table 1). Carbapenems, which have recently become a global public health problem, also showed a difference in resistance rates with temperature (*p* = 0.047). These results suggest that local temperature affects the antibiotic resistance rate of *K. pneumoniae*.

### 3.2. Genetic Characteristics of bla_KPC_- and bla_NDM_-Encoding K. pneumoniae

We selected 56 CP-KP isolates, including major domestic clones carrying *bla*_KPC_ (n = 29) and *bla*_NDM_ (n = 27). They mostly coexisted with extended-spectrum beta-lactamase (ESBL) genes, such as *bla*_CTX-M_. Isolates carrying *bla*_KPC_ most often harbored *bla*_TEM-1_ and *bla*_SHV-11_ together (37.9%), and isolates carrying *bla*_NDM_ harbored *bla*_TEM-1_, *bla*_SHV-11_, and *bla*_CTX-M-28_ (37.0%). Among the STs of isolates carrying *bla*_KPC_, ST258 (34.5%) was the most common, followed by ST307 (24.1%), ST392 (24.1%), and ST11 (10.3%, Table 2). ST14 (26.0%) and ST340 (26.0%) were the most common isolates carrying *bla*_NDM_, followed by ST1061 (22.2%), ST307 (7.4%), and ST11 (7.4%, Table 3). Among plasmids carrying the carbapenemase gene, *bla*_KPC_ belonged to a variety of incompatible groups. IncF was predominant (58.7%), followed by IncX3 (13.8%), IncN (13.8%), and IncH (10.3%). Most *bla*_NDM_-carrying plasmids belonged to the IncX3 group (77.8%), along with incompatible groups, such as IncF (IncFII and IncFIB) and IncHI.

### 3.3. Effect of Temperature on Conjugation of Plasmids Carrying bla_KPC_ and bla_NDM_

**Figure 1:**
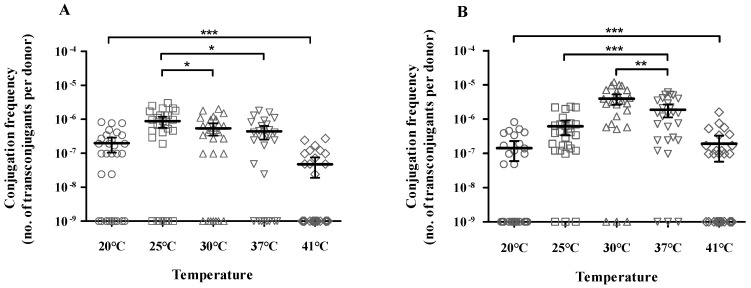
Conjugation efficiency according to temperature. (**A**) *bla*_KPC_- and (**B**) *bla*_NDM_-carrying *K. pneumoniae.* Each data point represents an individual. Each bar indicates the average value for a group, and error bars represent the 95% confidence interval for the ratio. The significance of differences between the temperature groups is shown as * *p* < 0.05, ** *p* < 0.01, and *** *p* < 0.001.

Measurement of the conjugation efficiency of the 56 CP-KP isolates revealed that most strains (82.1%) delivered a plasmid containing the carbapenemase gene to the recipient strain, except for 10 isolates. The carbapenemase gene-harboring plasmids were conjugated using *E. coli* J53 as a recipient at frequencies ranging from 10^−6^ to 10^−8^ transconjugants/donor. The conjugation efficiency of the plasmids carrying *bla*_KPC_ was (3.2 ± 2.5) × 10^−7^ at 20 °C, (1.1 ± 0.7) × 10^−6^ at 25 °C, (7.1 ± 5.3) × 10^−7^ at 30 °C, (6.4 ± 4.8) × 10^−7^ at 37 °C, and (1.1 ± 0.8) × 10^−7^ at 41 °C (Table 2 and Supplementary Table S1). The conjugation efficiency of the *bla*_NDM_-carrying plasmids was (2.7 ± 2.2) × 10^−7^ at 20 °C, (7.0 ± 7.3) × 10^−7^ at 25 °C, (4.5 ± 3.3) × 10^−6^ at 30 °C, (2.1 ± 1.9) × 10^−6^ at 37 °C, and (3.4 ± 4.0) × 10^−7^ at 41 °C (Table 3 and Supplementary Table S2). The optimal conjugation temperature for *bla*_KPC_-carrying strains was 25 °C (*p* = 0.030), and the conjugation frequencies did not vary at 30 °C and 37 °C (*p* = 0.077, Figure 1A). On the contrary, the *bla*_NDM_-carrying strains had the highest conjugation frequency at 30 °C (*p* = 0.007) and were more efficient at 37 °C than at 25 °C (*p* < 0.001, Figure 1B). These findings indicated that the conjugation frequency of the carbapenemase gene-harboring plasmid was not related to specific STs.

### 3.4. Stability of bla_KPC_- and bla_NDM_-Carrying Plasmids According to Temperature

**Figure 2:**
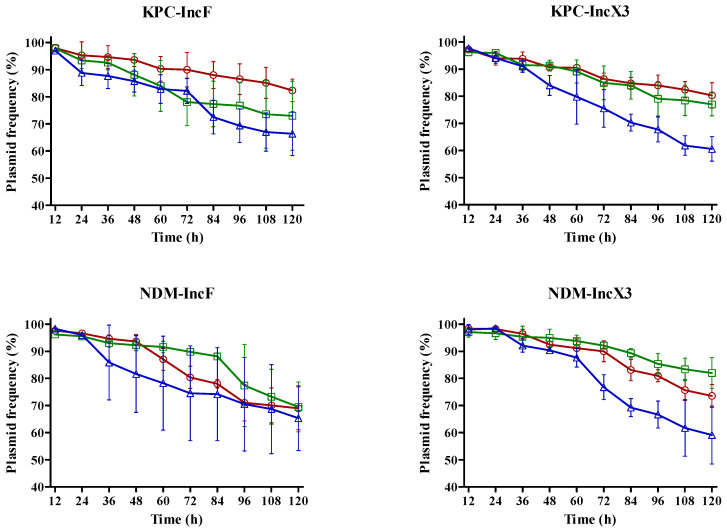
Impact of temperature on plasmid stability. Lines and symbols indicate values at each temperature: 25 °C, red; 30 °C, green; and 37 °C, blue. Error bars represent standard deviation.

Almost 60% of *bla*_KPC_- and *bla*_NDM_-encoding plasmids were maintained until the 10th passage (Figure 2). The *bla*_KPC_- and *bla*_NDM_-encoding plasmids showed high stability at 25 °C and 30 °C, respectively. *bla*_KPC_-IncF was more stable at 25 °C than at 30 °C (*p* = 0.032) and 37 °C (*p* = 0.047). The plasmid stability of *bla*_KPC_-IncX3 was the lowest at 37 °C (*p* = 0.047) and higher at 25 °C than at 30 °C but with no significant difference (*p* = 0.057). *bla*_NDM_-IncF was very stable at 30 °C until the 7th passage; however, no significant difference in plasmid stability was observed according to temperature after this passage. The plasmid stability of *bla*_NDM_-IncX3 was the highest at 30 °C and the lowest at 37 °C (*p* = 0.049), while no significant difference was observed between 30 °C and 25 °C (*p* = 0.387). As shown in Figure 1 and Figure 2, the conjugation frequency and stability of *bla*_KPC_- and *bla*_NDM_-encoding plasmids are dependent on temperature; in particular, the preferred temperature is different.

## 4. Discussion

A recent study on the distribution of antibiotic resistance in the USA revealed that increases in local temperature and population density were associated with increased antibiotic resistance [3]. The study showed that a 10 °C temperature rise was associated with a 4.2% and 2.2% increase in antibiotic resistance for *E. coli* and *K. pneumoniae*, respectively. Li et al. [16] reported that a 1 °C increase in the average regional temperature in China was associated with a 1.14- and 1.06-fold increase in carbapenem-resistant *K. pneumoniae* and *Pseudomonas aeruginosa* prevalence, respectively. Magnano San Lio et al. [17] also highlighted a close association between AMR and increasing temperatures as a consequence of the climate crisis. In contrast, another study found no seasonal variation in *K. pneumoniae* bloodstream infection rates or any association with average temperature [18]. Our study, unlike those from the United States, did not show a proportional increase in antibiotic resistance rates as regional temperatures rose. Nevertheless, the antibiotic resistance rate of *K. pneumoniae* in Korea exhibited significant differences depending on the regional temperature range over 5 years, suggesting that local temperature changes could affect the rate of antibiotic resistance in a specific region. To the best of our knowledge, this is the first study to describe the relationship between antibiotic resistance and local temperature in *K. pneumoniae* isolates from South Korea.

According to data from the KDCA, as of 2021, *K. pneumoniae* was the most common cause of CRE infections in Korea (68.6%). The proportion of CPE that affect the spread of CRE infections has increased from 2019 (57.8%) to 2021 (63.4%), of which more than 90% are KPC (76.2%) and NDM (19.7%) types [19]. The carbapenemase gene is usually located on a plasmid, and horizontal gene transfer contributes substantially to the spread of clones in epidemic CPE to plasmids containing carbapenemase genes [14,20]. 

Among the incompatible plasmid groups associated with carbapenemase genes in *Enterobacterales* [21], the *bla*_KPC_ gene is harbored by IncF, IncI2, IncX, IncA/C, IncR, and ColE1 [22], whereas *bla*_NDM_ is mainly located on plasmids of the IncX3 type [14]. The IncX3 plasmid transports and spreads carbapenemase genes (*bla*_NDM_, *bla*_KPC_, and *bla*_OXA-48_-like), particularly *bla*_NDM_ [23]. In our study, IncF was dominant for the *bla*_KPC_ gene, and IncX3 was predominant for *bla*_NDM_ (Table 2 and Table 3). IncF plasmids have been shown to participate in the global spread of various antibiotic resistance genes, accounting for nearly 40% of the plasmid-based carbapenemases [21]. *bla*_NDM_ is often located on the IncX3 plasmid and is considered the primary vehicle for *bla*_NDM_ transmission. The IncX3 plasmid is highly stable, with a low fitness cost and conjugation efficiency, which can facilitate the rapid and dominant dissemination of antibiotic resistance genes.

As shown in Tables S1 and S2, we revealed that the ST types of the KPC- and NDM-producing isolates were diverse. KPC and NDM have six and seven well-known ST types worldwide, including ST258 and ST11, respectively. The carbapenemase-encoding *bla*_KPC_-harboring plasmid is known worldwide to be primarily associated with clonal group 258 (CG258), which includes ST258, ST11, ST340, and ST512. ST258 is the predominant clone observed in European countries and the United States, and ST11, a single locus variant of ST258, is a widespread clonal type in Asia (especially China) [22]. Various ST strains have different conjugation frequencies [23]. In our study, all ST types showed active conjugation frequencies of between 10^−8^ and 10^−6^.

Several recent studies have reported that plasmid transfer is activated at a specific temperature, suggesting that the spread of antimicrobial-resistant bacteria may be temperature-related [13,14]. Additionally, certain replicon types have been reported to be affected by temperature [13,15,24,25], and some plasmids are conjugable only at specific temperatures [14]. The *bla*_NDM-1_-carrying IncA/C plasmid had the highest transfer rate at 25 °C or 30 °C, and the transfer efficiency of the IncH1 plasmid was optimal at 22–30 °C [26]. Our results showed that the *bla*_KPC_- and *bla*_NDM_-carrying plasmids were best transferred at 25 °C for IncF and 30 °C for IncX3, respectively, regardless of the ST type. According to Wang et al. (2018), IncX3 plasmids had similar or higher conjugation frequencies than IncFII plasmids at 30 °C and 37 °C, suggesting that the plasmid replicon types are associated with temperature in the horizontal transfer of antibiotic resistance genes [13]. IncX3 plasmids can be transferred between various Enterobacterial species over a wide temperature range [23]. Previous studies reported higher frequencies of plasmid delivery to recipients at 30 °C than at 25 °C or 37 °C [24,27] and that a temperature of 30 °C increases conjugation [25]. In contrast, another report showed that the IncX3 plasmid has a higher conjugation frequency, higher stability, and lower fitness cost at 37 °C [15,23]. However, the characteristics of the incompatible plasmid group alone cannot sufficiently explain why the optimized transfer temperatures varied between KPC and NDM.

KPC-producing *K. pneumoniae* was first reported in North Carolina, USA, in 1996 [28]; NDM-producing *K. pneumoniae* was detected in New Delhi, India, in 2008 [29] and then spread worldwide to become the most common carbapenemase. Despite the worldwide prevalence of KPC and NDM, the incidence of carbapenemases varies both geographically and regionally. In Korea, Spain, and Italy, these two types of carbapenemases are frequently reported [22]. All three countries are characterized by seasonal climates, with large intra-annual temperature ranges (summer and winter temperatures are often above and below 25 °C and 0 °C, respectively). The varying occurrence of specific CPE according to regional temperature is consistent with our results, in which KPC-producing strains showed a high conjugation frequency at 25 °C and NDM-producing strains at 30 °C. These findings further confirm that local temperature is critical for horizontal gene transfer in *Enterobacterales* [27]. Considering the higher conjugation frequency at temperatures lower than 37 °C, environmental transfer may be more important than in the intestine, even for *Enterobacterales* [27]. This indicates that the spread of genes involved in antibiotic resistance, including carbapenemases, may increase with increasing temperatures. 

Our study has some limitations. First, we did not analyze the incidence of infection because data on the total number of hospital patients were not included. Second, we did not consider variables other than temperature (such as humidity). Finally, it was difficult to confirm whether increases in temperature and rates of antibiotic resistance were proportional because of the brief period of data collection and small sample size. Nevertheless, temperature was clearly related to antibiotic resistance and resistance gene transfer.

## 5. Conclusions

The present study provides insights into the relationship between local temperature and antibiotic resistance rates in *K. pneumoniae* isolates from South Korea. The findings demonstrate that the resistance rate of *K. pneumoniae* in Korea differed significantly across temperature ranges based on local temperature. The study also revealed clear differences in the transfer of plasmids carrying the carbapenemase gene depending on temperature, with KPC- and NDM-producing strains showing high conjugation frequencies at 25 °C and 30 °C, respectively, which may also influence the global spread of CPE. These findings suggest that environmental factors, particularly local temperature, play a crucial role in the spread of antibiotic resistance genes, including carbapenemase genes.

## Figures and Tables

**Table 1:** Antibiotic-resistant *K. pneumoniae* isolates according to temperature over 5 years.

Antibiotics		No. of Resistant Isolates (%)				
Class	Drug	<0 °C	0–10 °C	10–20 °C	≥20 °C	*X* ^2^
		(N = 795)	(N = 2644)	(N = 2928)	(N = 3614)	
Carbapenems	Meropenem	9 (1.1)	37 (1.4)	61 (2.1)	78 (2.2)	0.047 *
Penicillins	Piperacillin	371 (46.7)	1125 (42.5)	1218 (41.6)	1611 (44.6)	0.018 *
β-lactam-lactamase inhibitors	Ampicillin-sulbactam	345 (43.4)	1000 (37.8)	1100 (37.6)	1477 (40.9)	0.001 *
Cephems	Cefotaxime	307 (38.6)	895 (33.9)	996 (34.0)	1316 (36.4)	0.016 *
	Ceftazidime	252 (31.7)	710 (26.9)	831 (28.4)	1076 (29.8)	0.018 *
	Cefepime	291 (36.6)	824 (31.2)	929 (31.7)	1221 (33.8)	0.009 *
	Cefoxitin	95 (11.9)	286 (10.8)	307 (10.5)	427 (11.8)	0.293
Monobactams	Aztreonam	263 (33.1)	737 (27.9)	871 (29.7)	1126 (31.2)	0.009 *
Aminoglycosides	Gentamicin	152 (19.1)	511 (19.3)	536 (18.3)	716 (19.8)	0.492
Fluoroquinolones	Ciprofloxacin	287 (36.1)	828 (31.3)	923 (31.5)	1223 (33.8)	0.015 *

* *p* < 0.05.

**Table 2:** Genetic characteristics and conjugation efficiency of *bla*_KPC_-carrying *K. pneumoniae*.

Pathogens	MLST Type	Replicon Type	Conjugation Efficiency	Pathogens	MLST Type	Replicon Type	Conjugation Efficiency
KP-KPC-01	ST258	IncFII	(9.0 ± 0.6) × 10^−7^	KP-KPC-15	ST101	IncFIIK	(3.3 ± 0.3) × 10^−7^
KP-KPC-02	ST258	IncFII	(6.5 ± 0.4) × 10^−7^	KP-KPC-16	ST307	IncFIB	(4.1 ± 0.3) × 10^−7^
KP-KPC-03	ST258	IncFIIK	(1.0 ± 0.5) × 10^−7^	KP-KPC-18	ST307	IncN	(14.8 ± 0.4) × 10^−7^
KP-KPC-04	ST258	IncFIIK	(4.8 ± 0.8) × 10^−7^	KP-KPC-19	ST307	IncN	(2.6 ± 0.5) × 10^−7^
KP-KPC-05	ST258	IncX3	(7.8 ± 0.6) × 10^−7^	KP-KPC-21	ST307	IncN	(1.1 ± 0.2) × 10^−6^
KP-KPC-06	ST258	IncX3	(7.0 ± 1.4) × 10^−7^	KP-KPC-23	ST307	IncFIB	NT
KP-KPC-07	ST258	IncX3	(6.8 ± 3.9) × 10^−8^	KP-KPC-25	ST307	IncFIB	NT
KP-KPC-08	ST258	IncFIB	NT	KP-KPC-26	ST307	IncHI1	NT
KP-KPC-10	ST258	IncX3	(2.1 ± 0.3) × 10^−7^	KP-KPC-17	ST392	IncHI2	(3.5 ± 0.3) × 10^−7^
KP-KPC-13	ST258	IncFIIK	(4.4 ± 0.3) × 10^−7^	KP-KPC-20	ST392	IncFIIK	(3.4 ± 0.3) × 10^−8^
KP-KPC-09	ST273	IncN	(1.0 ± 0.7) × 10^−6^	KP-KPC-22	ST392	IncFIB	(2.0 ± 0.6) × 10^−7^
KP-KPC-11	ST11	IncHI2	(2.2 ± 0.4) × 10^−7^	KP-KPC-24	ST392	IncFIB	(4.8 ± 1.1) × 10^−7^
KP-KPC-12	ST11	IncL	(3.9 ± 0.3) × 10^−7^	KP-KPC-27	ST392	IncFIIK	NT
KP-KPC-14	ST11	IncFIB	(1.3 ± 0.1) × 10^−6^	KP-KPC-28	ST392	IncFIB	NT
				KP-KPC-29	ST392	IncFIIK	NT

NT: not transferred.

**Table 3:** Genetic characteristics and conjugation efficiency of *bla*_NDM_-carrying *K. pneumoniae*.

Pathogens	MLST Type	Replicon Type	Conjugation Efficiency	Pathogens	MLST Type	Replicon Type	Conjugation Efficiency
KP-NDM-01	ST340	IncX3	(3.7 ± 1.4) × 10^−6^	KP-NDM-16	ST1061	IncX3	(1.1 ± 0.1) × 10^−6^
KP-NDM-02	ST340	IncX3	(1.7 ± 0.9) × 10^−8^	KP-NDM-19	ST1061	IncA/C	(1.8 ± 1.2) × 10^−7^
KP-NDM-03	ST340	IncX3	NT	KP-NDM-08	ST14	IncX2	(3.6 ± 1.5) × 10^−6^
KP-NDM-04	ST340	IncX3	(7.5 ± 5.9) × 10^−7^	KP-NDM-14	ST14	IncX3	(3.6 ± 0.8) × 10^−6^
KP-NDM-05	ST340	IncX3	NT	KP-NDM-17	ST14	IncX3	(1.7 ± 0.6) × 10^−6^
KP-NDM-06	ST340	IncX3	NT	KP-NDM-18	ST14	IncX3	(1.8 ± 0.4) × 10^−6^
KP-NDM-07	ST340	IncFIB	(2.1 ± 0.5) × 10^−7^	KP-NDM-20	ST14	IncX3	(1.8 ± 0.6) × 10^−6^
KP-NDM-09	ST11	IncX3	(2.3 ± 1.1) × 10^−6^	KP-NDM-21	ST14	IncX3	(1.4 ± 0.2) × 10^−6^
KP-NDM-10	ST11	IncX3	(3.5 ± 1.1) × 10^−6^	KP-NDM-22	ST14	IncHI	(2.1 ± 0.8) × 10^−6^
KP-NDM-11	ST1061	IncX3	(1.3 ± 0.6) × 10^−6^	KP-NDM-23	ST307	IncX3	(5.7 ± 2.3) × 10^−7^
KP-NDM-12	ST1061	IncX3	(8.5 ± 3.9) × 10^−7^	KP-NDM-24	ST307	IncX3	(1.0 ± 0.4) × 10^−6^
KP-NDM-13	ST1061	IncX3	(9.9 ± 4.1) × 10^−7^	KP-NDM-25	ST147	IncFII	(4.9 ± 0.4) × 10^−7^
KP-NDM-15	ST1061	IncA/C	(2.2 ± 1.9) × 10^−7^	KP-NDM-27	ST147	IncX3	(2.8 ± 0.5) × 10^−6^
				KP-NDM-26	ST789	IncX3	(8.4 ± 2.2) × 10^−7^

NT: not transferred.

## Data Availability

All data generated or analyzed during this study are included in this published article and its Supplementary Information files.

## Supplementary Materials

The following supporting information can be downloaded at: https://www.mdpi.com/article/10.3390/microorganisms12030454/s1, Figure S1: Flowchart of plasmid transfer by bacterial conjugation in this study; Table S1: Conjugation efficiency by temperature of *bla*_KPC_ carrying *K. pneumoniae* in this study; Table S2: Conjugation efficiency by temperature of *bla*_NDM_ carrying *K. pneumoniae* in this study.
