# Supplementary material for: Effect of Temperature on Carbapenemase-Encoding Plasmid Transfer in Klebsiella pneumoniae
Source: Microorganisms. 2024 Feb 23;12(3):454. doi: 10.3390/microorganisms12030454 (PMC10972239; doi:10.3390/microorganisms12030454)
Supplement: Supplementary file 1 [file microorganisms-12-00454-s001.zip › microorganisms-2838981-supplementary.pdf]

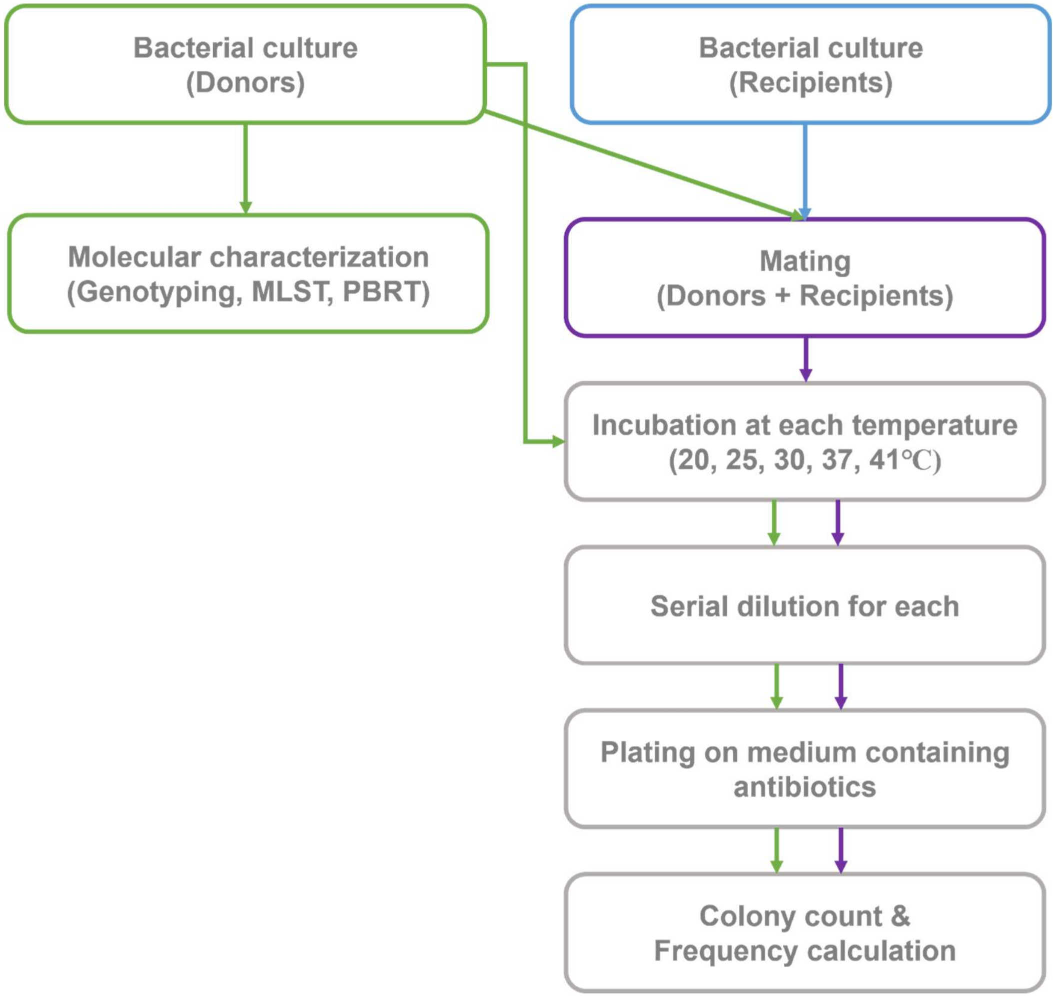

Supplementary Figure S1. Flowchart of plasmid transfer by bacterial conjugation in this study.

Supplementary Table S1. Conjugation efficiency by temperature of *bla*$_{KPC}$ carrying *K. pneumoniae* in this study.

Pathogens	Conjugation Efficiency				
	20℃	25℃	30℃	37℃	41℃
KP-KPC-01	(7.7 ± 0.4) X 10^{-7}	(18.4 ± 0.4) X 10^{-7}	(1.1 ± 0.1) X 10^{-6}	(7.0 ± 0.4) X 10^{-7}	(9.7 ± 4.2) X 10^{-8}
KP-KPC-02	(7.7 ± 0.4) X 10^{-7}	(13.3 ± 0.4) X 10^{-7}	(6.3 ± 0.4) X 10^{-7}	(4.8 ± 0.4) X 10^{-7}	(4.8 ± 4.2) X 10^{-8}
KP-KPC-03	(9.7 ± 4.2) X 10^{-7}	(2.9 ± 1.3) X 10^{-7}	(9.7 ± 4.2) X 10^{-8}	(2.4 ± 4.2) X 10^{-8}	NT
KP-KPC-04	(9.7 ± 4.2) X 10^{-7}	(12.6 ± 0.4) X 10^{-7}	(7.0 ± 2.2) X 10^{-7}	(3.4 ± 0.4) X 10^{-7}	(2.4 ± 0.4) X 10^{-7}
KP-KPC-05	(1.7 ± 0.4) X 10^{-7}	(16.9 ± 0.4) X 10^{-7}	(9.9 ± 0.4) X 10^{-7}	(9.2 ± 0.4) X 10^{-7}	(1.2 ± 1.1) X 10^{-7}
KP-KPC-06	(9.7 ± 4.2) X 10^{-7}	(1.3 ± 0.4) X 10^{-6}	(9.7 ± 0.8) X 10^{-7}	(10.6 ± 0.4) X 10^{-7}	(1.0 ± 1.1) X 10^{-7}
KP-KPC-07	NT	(1.9 ± 1.1) X 10^{-7}	(9.7 ± 4.2) X 10^{-8}	(4.8 ± 4.2) X 10^{-8}	NT
KP-KPC-08	NT	NT	NT	NT	NT
KP-KPC-10	(4.8 ± 0.4) X 10^{-7}	(4.6 ± 0.4) X 10^{-7}	(9.7 ± 4.2) X 10^{-8}	NT	NT
KP-KPC-13	NT	(8.9 ± 0.4) X 10^{-7}	(6.3 ± 0.4) X 10^{-7}	(6.3 ± 0.4) X 10^{-7}	(4.8 ± 4.2) X 10^{-8}
KP-KPC-09	(8.2 ± 0.4) X 10^{-7}	(2.1 ± 0.4) X 10^{-6}	(1.2 ± 0.2) X 10^{-6}	(8.2 ± 0.4) X 10^{-7}	(1.2 ± 1.1) X 10^{-7}
KP-KPC-11	(1.9 ± 0.4) X 10^{-7}	(4.1 ± 0.8) X 10^{-7}	(3.4 ± 0.4) X 10^{-7}	(1.7 ± 0.4) X 10^{-7}	NT
KP-KPC-12	(3.4 ± 0.4) X 10^{-7}	(9.7 ± 0.4) X 10^{-7}	(4.1 ± 0.4) X 10^{-7}	(2.4 ± 0.4) X 10^{-7}	NT
KP-KPC-14	(2.4 ± 0.4) X 10^{-7}	(2.5 ± 0.4) X 10^{-6}	(17.6 ± 0.4) X 10^{-7}	(16.4 ± 0.4) X 10^{-8}	(2.4 ± 1.1) X 10^{-7}
KP-KPC-15	(9.7 ± 4.2) X 10^{-7}	(6.8 ± 0.4) X 10^{-7}	(4.8 ± 0.4) X 10^{-7}	(4.1 ± 0.4) X 10^{-8}	NT
KP-KPC-16	(1.9 ± 0.4) X 10^{-7}	(9.7 ± 0.4) X 10^{-7}	(5.6 ± 0.4) X 10^{-7}	(3.4 ± 0.4) X 10^{-7}	NT
KP-KPC-18	(3.4 ± 0.4) X 10^{-7}	(30.1 ± 0.4) X 10^{-7}	(20.0 ± 0.4) X 10^{-7}	(17.9 ± 0.4) X 10^{-7}	(2.7 ± 0.4) X 10^{-7}
KP-KPC-19	(2.4 ± 4.2) X 10^{-7}	(5.3 ± 0.4) X 10^{-7}	(2.7 ± 0.4) X 10^{-7}	(4.1 ± 0.4) X 10^{-7}	(4.8 ± 8.4) X 10^{-8}

KP-KPC-21	$(4.1 \pm 0.4) \times 10^{-7}$	$(2.1 \pm 0.4) \times 10^{-6}$	$(1.5 \pm 0.2) \times 10^{-6}$	$(17.9 \pm 0.4) \times 10^{-7}$	$(1.7 \pm 0.8) \times 10^{-7}$
KP-KPC-23	NT	NT	NT	NT	NT
KP-KPC-25	NT	NT	NT	NT	NT
KP-KPC-26	NT	NT	NT	NT	NT
KP-KPC-17	$(2.7 \pm 0.4) \times 10^{-7}$	$(6.8 \pm 0.4) \times 10^{-7}$	$(4.1 \pm 0.4) \times 10^{-7}$	$(4.1 \pm 0.4) \times 10^{-7}$	NT
KP-KPC-20	$(9.7 \pm 4.2) \times 10^{-7}$	$(6.8 \pm 0.4) \times 10^{-7}$	$(4.8 \pm 0.4) \times 10^{-7}$	$(4.6 \pm 0.4) \times 10^{-7}$	NT
KP-KPC-22	$(2.4 \pm 4.2) \times 10^{-7}$	$(4.6 \pm 1.7) \times 10^{-7}$	$(2.7 \pm 0.4) \times 10^{-7}$	$(2.4 \pm 0.4) \times 10^{-7}$	NT
KP-KPC-24	$(1.9 \pm 0.4) \times 10^{-7}$	$(1.1 \pm 0.2) \times 10^{-6}$	$(6.8 \pm 2.1) \times 10^{-7}$	$(3.4 \pm 0.4) \times 10^{-8}$	$(7.2 \pm 7.2) \times 10^{-7}$
KP-KPC-27	NT	NT	NT	NT	NT
KP-KPC-28	NT	NT	NT	NT	NT
KP-KPC-29	NT	NT	NT	NT	NT

* NT: Not Transferred

Supplementary Table S2. Conjugation efficiency by temperature of *bla*$_{NDM}$ carrying *K. pneumoniae* in this study.

Pathogens	Conjugation Efficiency				
	20℃	25℃	30℃	37℃	41℃
KP-NDM-01	(5.1 ± 2.6) X 10^{-7}	(2.2 ± 0.7) X 10^{-6}	(9.5 ± 2.9) X 10^{-6}	(6.1 ± 2.8) X 10^{-6}	(9.7 ± 8.4) X 10^{-8}
KP-NDM-02	(4.8 ± 4.2) X 10^{-8}	(1.4 ± 0.7) X 10^{-7}	(5.8 ± 2.6) X 10^{-7}	(9.7 ± 8.4) X 10^{-8}	NT
KP-NDM-03	NT	NT	NT	NT	NT
KP-NDM-04	NT	(2.9 ± 2.5) X 10^{-7}	(2.7 ± 2.2) X 10^{-6}	(7.2 ± 5.0) X 10^{-7}	NT
KP-NDM-05	NT	NT	NT	NT	NT
KP-NDM-06	NT	NT	NT	NT	NT
KP-NDM-07	NT	(1.2 ± 0.4) X 10^{-7}	(5.8 ± 1.3) X 10^{-7}	(2.4 ± 0.4) X 10^{-7}	(1.2 ± 0.4) X 10^{-7}
KP-NDM-09	(3.9 ± 0.8) X 10^{-7}	(1.1 ± 0.5) X 10^{-6}	(7.1 ± 4.0) X 10^{-6}	(2.2 ± 0.8) X 10^{-6}	(5.3 ± 3.3) X 10^{-7}
KP-NDM-10	NT	(4.1 ± 0.4) X 10^{-7}	(1.0 ± 0.3) X 10^{-5}	(5.2 ± 1.1) X 10^{-6}	(1.6 ± 1.3) X 10^{-6}
KP-NDM-11	(9.7 ± 4.2) X 10^{-8}	(3.9 ± 0.8) X 10^{-7}	(4.2 ± 2.5) X 10^{-6}	(1.5 ± 0.5) X 10^{-6}	(1.7 ± 0.4) X 10^{-7}
KP-NDM-12	NT	(1.9 ± 0.4) X 10^{-7}	(2.8 ± 1.6) X 10^{-6}	(1.2 ± 0.3) X 10^{-6}	NT
KP-NDM-13	NT	(3.4 ± 1.7) X 10^{-7}	(2.9 ± 1.4) X 10^{-6}	(1.6 ± 0.5) X 10^{-6}	(9.7 ± 4.2) X 10^{-8}
KP-NDM-15	NT	(1.2 ± 1.1) X 10^{-7}	(7.2 ± 6.4) X 10^{-7}	(2.4 ± 2.1) X 10^{-7}	NT
KP-NDM-16	(1.7 ± 0.8) X 10^{-7}	(1.2 ± 0.4) X 10^{-7}	(3.6 ± 0.1) X 10^{-6}	(1.5 ± 0.2) X 10^{-6}	(1.7 ± 0.4) X 10^{-7}
KP-NDM-19	NT	(1.7 ± 0.8) X 10^{-7}	(5.1 ± 4.4) X 10^{-7}	(2.4 ± 0.8) X 10^{-7}	NT

KP-NDM-08	$(4.1 \pm 3.3) \times 10^{-7}$	$(2.0 \pm 0.5) \times 10^{-6}$	$(9.7 \pm 3.5) \times 10^{-6}$	$(5.2 \pm 2.3) \times 10^{-6}$	$(7.5 \pm 6.5) \times 10^{-7}$
KP-NDM-14	$(3.4 \pm 0.4) \times 10^{-7}$	$(7.0 \pm 1.7) \times 10^{-7}$	$(1.2 \pm 0.3) \times 10^{-5}$	$(5.0 \pm 0.3) \times 10^{-6}$	$(9.7 \pm 4.2) \times 10^{-8}$
KP-NDM-17	$(4.8 \pm 4.2) \times 10^{-8}$	$(7.0 \pm 0.4) \times 10^{-7}$	$(5.2 \pm 2.1) \times 10^{-6}$	$(2.1 \pm 0.5) \times 10^{-6}$	$(3.4 \pm 0.4) \times 10^{-7}$
KP-NDM-18	$(1.9 \pm 0.4) \times 10^{-7}$	$(6.5 \pm 1.3) \times 10^{-7}$	$(3.4 \pm 0.5) \times 10^{-6}$	$(4.1 \pm 0.8) \times 10^{-6}$	$(5.6 \pm 2.9) \times 10^{-7}$
KP-NDM-20	$(8.2 \pm 2.1) \times 10^{-7}$	$(4.3 \pm 1.3) \times 10^{-7}$	$(4.7 \pm 1.8) \times 10^{-6}$	$(2.9 \pm 0.8) \times 10^{-6}$	NT
KP-NDM-21	NT	$(2.2 \pm 0.2) \times 10^{-6}$	$(3.9 \pm 0.8) \times 10^{-6}$	$(6.0 \pm 2.1) \times 10^{-7}$	NT
KP-NDM-22	NT	$(5.3 \pm 3.3) \times 10^{-7}$	$(6.4 \pm 2.8) \times 10^{-6}$	$(3.5 \pm 1.1) \times 10^{-6}$	NT
KP-NDM-23	$(1.4 \pm 1.3) \times 10^{-7}$	$(9.7 \pm 8.4) \times 10^{-8}$	$(1.2 \pm 0.5) \times 10^{-6}$	$(1.4 \pm 0.4) \times 10^{-6}$	NT
KP-NDM-24	NT	$(7.5 \pm 0.8) \times 10^{-7}$	$(3.4\pm 1.3) \times 10^{-6}$	$(7.5 \pm 3.3) \times 10^{-7}$	$(9.7 \pm 4.2) \times 10^{-8}$
KP-NDM-25	$(1.2 \pm 0.4) \times 10^{-7}$	$(1.7 \pm 0.4) \times 10^{-7}$	$(17.6 \pm 0.4) \times 10^{-7}$	$(2.9 \pm 1.3) \times 10^{-7}$	$(9.7 \pm 4.2) \times 10^{-8}$
KP-NDM-27	$(4.6 \pm 0.8) \times 10^{-7}$	$(23.4 \pm 0.8) \times 10^{-7}$	$(7.0 \pm 1.5) \times 10^{-6}$	$(4.0 \pm 0.8) \times 10^{-6}$	$(3.6 \pm 1.3) \times 10^{-7}$
KP-NDM-26	$(9.7 \pm 8.4) \times 10^{-8}$	$(6.3 \pm 0.4) \times 10^{-7}$	$(3.2 \pm 0.8) \times 10^{-6}$	$(1.2 \pm 0.8) \times 10^{-7}$	$(1.2 \pm 0.4) \times 10^{-7}$

* NT: Not Transferred
